# Knowledge Attitudes and Practices Regarding Malaria and HIV in People Living in Rural Ghana

**DOI:** 10.1002/hsr2.70833

**Published:** 2025-05-19

**Authors:** Felix Abekah Botchway, Cecilia Elorm Lekpor, Prince Agyeman, Ebenezer Krampah Aidoo, Jacob Apibilla Ayembilla, Micheal Appiah, Ahmed Mashud, Richard Kobina Dadzie Ephraim

**Affiliations:** ^1^ Department of Medical Laboratory Science Accra Technical University Accra Ghana; ^2^ Department of Pathology University of Ghana Medical School Accra Ghana; ^3^ Department of Medical Laboratory Science KAAF University College Accra Ghana; ^4^ Department of Medical Laboratory Science University of Cape Coast Cape Coast Ghana

**Keywords:** HIV, knowledge, attitudes, and practices, malaria

## Abstract

**Introduction:**

The realization of malaria and Human immunodeficiency virus (HIV) control in any community cannot be effective if the said community's knowledge and application of control mechanisms are not appropriately defined. However, knowledge, attitudes, and practices studies regarding malaria and human immunodeficiency virus (HIV) specifically in people living in rural Ghana are scant, and as such, minimal interventions focus on them. This study reports the results of individuals' knowledge, attitudes, and practices in rural Ghana.

**Methods:**

This cross‐sectional study involved 316 individuals who visited the daily OPD at the Shai‐Osudoku District Hospital. Responses from participants were recorded on a semi‐structured questionnaire. Data entry was done with Microsoft Office Excel 2010, analysis was performed using the Statistical Package for Social Sciences (SPSS), version 20.0, and the results were summarized using tables.

**Results:**

More than 94% of respondents in all categories indicated they knew the clinical signs/symptoms, transmission, and prevention of malaria. Although 66.0% of malaria‐negative respondents and 62.2% of malaria‐positive respondents preferred using mosquito nets to protect themselves, only 39.4% of malaria‐negative respondents and 32.8% of malaria‐positive respondents reported sleeping under a mosquito net the previous night. Mosquito coil as a malaria preventive method recorded the least preference with less than 7% in all categories. Over 94% of the respondents in all categories knew about HIV, yet misconceptions about transmission persisted. Only 50% of malaria‐negative respondents and 36.7% of malaria‐positive respondents had ever tested for HIV, while 17% of malaria‐negative respondents indicated they did not know where to get tested for HIV. Discriminatory attitudes were present in a considerable proportion of the respondents.

**Conclusion:**

The respondents demonstrated knowledge about HIV and malaria. Understanding the KAP about HIV and malaria in the general populations will help us in formulating strategies for prevention and treatment. Our study calls for continued and strengthened health education to bring change in knowledge regarding misconceptions about the mode of transmission of HIV and how to properly use insecticide‐treated nets.

## 1 Introduction

Malaria is a serious health challenge in most Sub‐Saharan Africa (SSA) countries of which Ghana is a part. In Sub‐Saharan Africa, it is estimated that more than 300 million people are at risk of malaria infection, thus accounting for about half of the worldwide burden [1]. Malaria is endemic in Ghana, with an incidence of 40% of all outpatient attendance [1]. *Plasmodium falciparum* constitutes more than 95.0% of the parasite species in Ghana. Though low‐level infections of *Plasmodium ovale* and *Plasmodium malariae* commonly occur as mixed infections with *Plasmodium falciparum*, however, *Plasmodium vivax* is yet to be reported [1, 2]. The current thrust of malaria vector control in Ghana is in the use of insecticide‐based interventions both at the individual and communal levels. Most national malaria control programs, including Ghana, put more emphasis on parasite and/or vector control, overlooking the target population's knowledge, attitudes, and practices in the transmission and control of the disease [3].

Acquired immune deficiency syndrome (AIDS) is caused by the human immunodeficiency virus (HIV) and remains a serious public health problem in Ghana and SSA. HIV in the mid‐1990s outshone both malaria and measles to become the second leading cause of child mortality in SSA [4]. In 2013, about 35 million persons were living with HIV (PLHIV) infection, while about 1.5 million deaths were recorded due to the disease globally. As of 2012, about 71% of persons living with HIV globally were in sub‐Saharan Africa [5].

In Ghana, the Human Immunodeficiency Virus (HIV) has established a low‐level generalized epidemic with mostly higher prevalence among certain populations and special groups. Ghana recorded a prevalence rate of 1.5% in 2013 [6]. Despite the low prevalence rate, efforts to react to the epidemic need to be sustained and scaled up to maintain a lower prevalence. The youth are more predisposed to HIV infection and other sexually transmitted diseases due to a lack of correct health information and access to adequate reproductive health services, their engagement in risky behaviors, economic exploitation, regional and national conflicts [5, 7]. The World Health Organization (W.H.O.), in 1990, declared that people with enough knowledge of HIV stood a better chance of preventing themselves from the disease than those without knowledge [4].

In communities where interaction between malaria and HIV is observed, it will be advantageous if these two are considered together in intervention programs [8]. These diseases are incriminated as a cause for a reduction in economic growth [9]. The socioeconomic impact of malaria in countries with intense transmission accounts for an average of 1.3% loss of their annual economic growth, leading to significant differences in Gross Domestic Product (GDP) between countries with and without malaria. Malaria has been projected to cost Africa more than US$12 billion every year in lost GDP. A difference in malaria patterns has been observed between HIV‐infected individuals and HIV‐negative individuals [8, 10]. To make things worse, to date, there is no vaccine against malaria or HIV.

HIV has been revealed to intensify the susceptibility and the severity of malaria. Nevertheless, the severity is increased in individuals without previously acquired partial immunity to malaria [8, 11, 12].

The realization of malaria and HIV control in any community cannot be effective if the said community's knowledge and application of control mechanisms are not appropriately defined. Thus, extensive studies of knowledge, attitude, and practice (KAP) are obligatory to make informed and shaped intervention policies in the fight against HIV and malaria in Ghana. However, KAP studies regarding malaria and HIV specifically in people living in rural Ghana are scant; and as such, very few of these interventions focus on them. This study reports the results of knowledge, attitudes, and practices of individuals visiting the Shai‐Osudoku district hospital in rural Ghana.

## 2 Methods

### 2.1 Study Design/Site

This was a cross‐sectional study, designed to assess the knowledge, attitude, and practice of the residents in Dodowa and its surrounding villages in the Shai‐Osudoku District who visited the daily OPD at the Shai‐Osudoku District Hospital. The Shai‐Osudoku District, situated in the Greater Accra Region, is home to rural and peri‐urban populations with diverse socioeconomic backgrounds. Considering the growing population, Shai‐Osudoku is a critical healthcare hub for residents, including vulnerable groups such as the elderly, pregnant women, and children. The district hospital serves as the primary referral center and provides essential services to this population, making it an ideal setting for studying disease patterns and health interventions.

### 2.2 Study Population

The study population consisted of 360 individuals between the ages of 17 and 49 years who visited the daily OPD at the Shai‐Osudoku District Hospital.

### 2.3 Data Collection Tools and Techniques

A convenient sampling technique was used to sample both men and women, and they were interviewed. Data were collected using structured, pre‐tested, and interviewer‐administered questionnaires. The questionnaire was prepared in English and then translated into the local dialect for the data collection. Each interview lasted approximately 15 min. The questionnaire included questions that address relevant socio‐demographic information about the respondents, questions directly assessing the knowledge, clinical signs/symptoms, transmission, and prevention of malaria and HIV; and questions about attitude towards PLHIV.

### 2.4 Data Analysis

Entry of data was done with Microsoft Office Excel 2010 (Microsoft Corporation, USA), analysis was performed using the Statistical Package for Social Sciences (SPSS), version 20.0, and the results were summarized using tables. Both qualitative and quantitative data were collected. Quantitative variables were summarized by median and interquartile range (IQR). For qualitative variables, the number and percentage of subjects in each category were given. The level of significance (α) at 0.05 was used for all tests of statistical significance.

## 3 Results

### 3.1 Sociodemographic Characteristics of Malaria‐Negative and Malaria‐Positive Respondents

Table 1 shows the association between the sociodemographic characteristics of participants, malaria‐ and malaria‐positive respondents. In both malaria‐negative and malaria‐positive respondents, female [67/94 (71.3%) and 75/119 (63.0%)] respectively outnumbered male respondents [27/94 (28.7%) and 44/119 (37.0%)]. The median age of malaria‐negative respondents was significantly higher than malaria‐positive respondents (*p* < 0.001). In both malaria‐negative and malaria‐positive cases, the majority of the respondents were senior high school leavers [29/94 (30.9%) and 43/119 (36.1%)] respectively. Most of the respondents were single in both malaria‐negative and malaria‐positive cases [43/94 (45.7%) and 61/119 (51.3%)], respectively.

**Table 1:** Sociodemographic characteristics of malaria‐negative and malaria‐positive respondents.

Characteristics	Malaria‐negative (*N* = 94)	Malaria‐positive (*N* = 119)	*p* value
Sex			
Male	27 (28.7%)	44 (37.0%)	0.205
Female	67 (71.3%)	75 (63.0%)	
Age (years)			
Median (IQR)	29 (22–35)	23 (17–31)	< 0.001
Education status			
No education	5 (5.3%)	3 (2.5%)	0.411
Primary/elementary school	10 (10.6%)	16 (13.5%)	
Junior high school	17 (18.1%)	27 (22.7%)	
Senior high school	29 (30.9%)	43 (36.1%)	
Tertiary	27 (28.7%)	22 (18.5%)	
Marital status			
Single	43 (45.7%)	61 (51.3%)	0.339
Married	5 (5.3%)	10 (8.40%)	
Widowed	5 (5.3%)	2 (1.7%)	
Divorced	—	1 (0.8%)	
Separated	35 (37.2%)	34 (28.6%)	

Abbreviation: IQR, interquartile range.

### 3.2 Malaria Knowledge and Prevention

Respondents' knowledge of malaria and its prevention are summarized in Table 2. Eighty‐nine (94.7%) of malaria‐negative respondents indicated that they knew about malaria, as against 112 (94.1%) malaria‐positive respondents. Mosquito nets were the most used preventive method by both malaria‐negative and malaria‐positive respondents [62/94 (60.0%) and 74/119 (62.2%)], respectively. Mosquito coils were the least used preventive method in both malaria‐negative and malaria‐positive respondents [6/94 (6.4%) and 6/119 (5.0%)], respectively. Most participants in both malaria‐negative and malaria‐positive cases had mosquito nets in their homes [61/94 (64.9%) and 78/119 (65.6%)], respectively. 61.7% and 56.3% of malaria‐negative and malaria‐positive respondents, respectively, used mosquito nets treated with insecticide. Some malaria‐negative and malaria‐positive respondents slept under a mosquito net the night before the interview.

**Table 2:** Malaria‐positive and malaria‐negative respondents' knowledge and prevention of malaria.

Characteristics	Malaria‐negative (*N* = 94)	Malaria‐positive (*N* = 119)	*p* value
Knowledge of malaria	89 (94.7%)	112 (94.1%)	0.859
Malaria prevention method used			
Mosquito coils	6 (6.4%)	6 (5.0%)	0.298
Spray	14 (14.9%)	21 (17.7%)	
Mosquito nets	62 (66.0%)	74 (62.2%)	
Mosquito net in the household	61 (64.9%)	78 (65.6%)	0.808
Mosquito net treated with insecticide	58 (61.7%)	67 (56.3%)	0.810
Used a mosquito net last night	37 (39.4%)	39 (32.8%)	0.575

### 3.3 Knowledge and Attitude Towards HIV Among Malaria‐Positive and Malaria‐Negative Respondents

Participants were asked questions to assess their knowledge of HIV directly. Eighty‐seven (92.6%) of malaria‐negative respondents and 108 (90.8%) malaria‐positive respondents indicated that they know about HIV. When asked more specifically, the majority of both respondents indicated that having one sex partner reduces the chance of getting HIV. However, some respondents, both malaria‐negative (81.9%) and malaria‐positive (79.8%), pointed out that the use of condoms prevents HIV infection. Few other malaria‐negative (2.1%) and malaria‐positive (5.9%) respondents indicated that HIV can be acquired by sharing food with an infected person. With regard to HIV status, some respondents [malaria‐negative (50.0%), malaria‐positive (36.7%)] had tested for HIV and knew their status. In comparison, 17.0% of malaria‐negative and 74.8% of malaria‐positive respondents knew a place to get tested for HIV. Few of the respondents indicated that they would buy food from a vendor if they knew the person had HIV. (Table 3)

**Table 3:** Knowledge and attitude of malaria‐negative and malaria‐positive respondents towards HIV.

Characteristics	Malaria‐negative (*N* = 94)	Malaria‐positive (*N* = 119)	*p* value
Knowledge of HIV	87 (92.6%)	108 (90.8%)	0.648
Having one sex partner reduces the chance of getting HIV	78 (83.0%)	87 (73.1%)	0.232
The use of condoms prevents HIV infection	77 (81.9%)	95 (79.8%)	0.911
HIV can be acquired by sharing food with an infected person	2 (2.1%)	7 (5.9%)	0.491
Tested for HIV	47 (50.0%)	46 (36.7%)	0.252
Know a place to get an HIV test	68 (17.0%)	89 (74.8%)	0.363
Will buy food from a vendor if they know the person has HIV	13 (13.8%)	17 (14.3%)	0.263

Abbreviation: HIV, human immunodeficiency virus.

### 3.4 The Association Between the Sociodemographic Characteristics of Malaria‐Positive and HIV‐Positive Respondents

Table 4 shows the association between the sociodemographic characteristics of malaria‐positive and HIV‐positive respondents. In both malaria‐positive and HIV‐positive respondents, females [75/119 (63%) and 67/103 (65.1%)], respectively, outnumbered males [44/119 (37.0%) and 36/103 (34.9%)]. The median age of HIV‐positive respondents was significantly higher than malaria‐positive respondents (*p* < 0.001). Forty‐three (36.1%) of malaria‐positive respondents were senior high school leavers, while 30 (29.1%) HIV‐positive respondents were junior high school leavers. There was a significant difference in marital status between malaria‐positive and HIV‐positive respondents (*p* < 0.001).

**Table 4:** Sociodemographic characteristics of malaria‐positive and HIV‐positive respondents.

Characteristics	Malaria‐positive (*N* = 119)	HIV‐positive (*N* = 103)	*p* value
Sex			
Male	44 (37.0%)	36 (34.9%)	0.754
Female	75 (63%)	67 (65.1%)	
Age (years)			
Median (IQR)	23 (17–31)	42 (30–49)	< 0.001
Education status			
No education	3 (2.5%)	14 (13.6%)	0.008
Primary/elementary school	16 (13.5%)	24 (23.3%)	
Junior high school	27 (22.7%)	30 (29.1%)	
Senior high school	43 (36.1%)	26 (25.2%)	
Tertiary	22 (18.5%)	7 (6.8%)	
Marital status			
Single	61 (51.3%)	37 (35.9%)	< 0.001
Married	10 (8.40%)	42 (40.8%)	
Widowed	2 (1.7%)	13 (12.6%)	
Divorced	1 (0.8%)	4 (3.9%)	
Separated	34 (28.6%)	5 (4.9%)	

Abbreviation: HIV, human immunodeficiency virus.

### 3.5 Malaria Knowledge and Prevention

Table 5 summarizes respondents' knowledge of malaria and its preventive methods. Most malaria‐positive and HIV‐positive respondents revealed they knew about malaria. Several preventive methods were expressed by the respondents, 62.2% and 81.6% of malaria‐positive and HIV‐positive respondents, respectively, preferred mosquito nets to other methods. Mosquito coil was the least preferred method by respondents. Amusingly, sleeping under insecticide‐treated mosquito nets accounted for the least with 56.3%, 63.1% by both malaria‐positive and HIV‐positive respondents. There was no significant difference between knowledge of malaria and preventive methods.

**Table 5:** Knowledge and prevention of respondents about malaria.

Characteristics	Malaria‐positive (*N* = 119)	HIV‐positive (*N* = 103)	*p* value
Knowledge of malaria	112 (94.1%)	100 (97.1%)	0.131
Malaria prevention method used			
Mosquito coils	6 (5.0%)	4 (3.9%)	0.008
Spray	21 (17.7%)	8 (7.8%)	
Mosquito nets	74 (62.2%)	84 (81.6%)	
Mosquito net in the household	78 (65.6%)	66 (64.1%)	0.475
Mosquito net treated with insecticide	67 (56.3%)	65 (63.1%)	0.308
Used a mosquito net last night	39 (32.8%)	39 (37.9%)	0.717

Abbreviation: HIV, human immunodeficiency virus.

### 3.6 HIV‐Positive and Malaria‐Positive Respondents' Knowledge About HIV

The respondents' knowledge of HIV is shown in Table 6. All the HIV+ respondents (100%) indicated that they know about HIV, as against 90.8% of malaria respondents. The majority of malaria‐positive (73.1%) and HIV‐positive respondents (92.2%) significantly stated that having one sex partner reduces the chance of getting HIV (*p* < 0.001). The percentage of malaria‐positive respondents (36.7%) who had tested for HIV was significantly lower than that of the 100% HIV‐positive respondents (*p* < 0.001). The proportion of HIV‐positive respondents (58.3%) who indicated that they would buy food from a vendor if they knew they were infected with HIV was significantly higher than that of malaria respondents (*p* < 0.001).

**Table 6:** HIV‐positive and malaria‐positive respondents' knowledge about HIV.

Characteristics	Malaria‐positive (*N* = 119)	HIV‐positive (*N* = 103)	*p* value
Knowledge of HIV	108 (90.8%)	103 (100%)	0.423
Having one sex partner reduces the chance of getting HIV	87 (73.1%)	95 (92.2%)	< 0.001
A condom prevents HIV infection	95 (79.8%)	88 (85.4%)	0.625
HIV can be acquired by sharing food with an infected person	7 (5.9%)	13 (12.6%)	0.064
Tested for HIV	46 (36.7%)	103 (100%)	< 0.001
Know a place to get an HIV test	89 (74.8%)	91 (88.4%)	0.025
Will buy food from a vendor if they know the person has HIV	17 (14.3%)	60 (58.3%)	< 0.001

Abbreviation: HIV, human immunodeficiency virus.

### 3.7 Sociodemographic Characteristics of HIV‐Positive and Malaria‐Negative Respondents

Table 7 shows the association between the sociodemographic characteristics of HIV‐positive and malaria‐negative respondents. Although statistically insignificant, the female respondents were more than the male respondents. The median age of HIV‐positive respondents was 42 years (30–49 years) which was significantly higher than among malaria‐negative respondents, 29 years (22–35 years) (*p* < 0.001). The smallest percentage of HIV‐positive respondents (6.8%) significantly had tertiary education, whilst the majority of malaria‐negative respondents (30.9%) were senior high school leavers (*p* < 0.001). A significant percentage of HIV+ respondents (40.8%) were married, whereas 45.7% of malaria‐negative respondents were single (*p* < 0.001). Even as 3.9% of HIV‐positive respondents were significantly divorced, none of the malaria‐negative respondents were divorced (*p* < 0.001).

**Table 7:** Sociodemographic characteristics of HIV‐positive and malaria‐negative respondents.

Characteristics	Malaria‐negative (*N* = 94)	HIV‐positive (*N* = 103)	*p* value
Sex			
Male	27 (28.7%)	36 (34.9%)	0.349
Female	67 (71.3%)	67 (65.1%)	
Age (years)			
Median (IQR)	29 (22–35)	42 (30–49)	< 0.001
Education status			
No education	5 (5.3%)	14 (13.6%)	< 0.001
Primary/elementary school	10 (10.6%)	24 (23.3%)	
Junior high school	17 (18.1%)	30 (29.1%)	
Senior high school	29 (30.9%)	26 (25.2%)	
Tertiary	27 (28.7%)	7 (6.8%)	
Marital status			
Single	43 (45.7%)	37 (35.9%)	< 0.001
Married	5 (5.3%)	42 (40.8%)	
Widowed	5 (5.3%)	13 (12.6%)	
Divorced	—	4 (3.9%)	
Separated	35 (37.2%)	5 (4.9%)	

Abbreviation: HIV, human immunodeficiency virus.

### 3.8 Malaria Knowledge and Prevention

Table 8 summarizes HIV‐positive and malaria‐negative respondents' knowledge of malaria and its preventive methods. In general, the majority of malaria‐negative (94.7%) and HIV‐positive respondents (97.1%) had significant knowledge of malaria (*p* < 0.001). Mosquito net usage as a malaria preventive method was significantly high in both malaria‐negative and HIV‐positive respondents (*p* = 0.003). A significantly higher percentage of the respondents indicated they had mosquito nets at home (*p* = 0.027).

**Table 8:** HIV‐positive and malaria‐negative respondents' knowledge of malaria and its preventive methods.

Characteristics	Malaria‐negative (*N* = 94)	HIV‐positive (*N* = 103)	*p* value
Knowledge of malaria	89 (94.7%)	100 (97.1%)	< 0.001
Malaria prevention method used			
Mosquito coils	6 (6.4%)	4 (3.9%)	0.003
Spray	14 (14.9%)	8 (7.8%)	
Mosquito nets	62 (66.0%)	84 (81.6%)	
Mosquito net in the household	61 (64.9%)	66 (64.1%)	0.027
Mosquito net treated with insecticide	58 (61.7%)	65 (63.1%)	0.241
Used a mosquito net last night	37 (39.4%)	39.37.9%)	0.193

Abbreviation: HIV, human immunodeficiency virus.

### 3.9 HIV‐Positive and Malaria‐Negative Respondents' Knowledge of HIV

Table 9 summarizes HIV‐positive and malaria‐negative respondents' knowledge about HIV. Unsurprisingly, 100% of HIV‐positive respondents and 92.6% of malaria‐negative respondents indicated a significant amount of knowledge about HIV (*p* = 0.004). Over 80% of all respondents indicated significantly that having one sex partner and the use of condoms reduces/prevents the transmission of the virus (*p* = 0.003). However, 2.1% of malaria‐negative and 12.6% of HIV‐positive respondents believed significantly that HIV can be acquired by sharing food with an infected person (*p* = 0.002). Whilst 100% of HIV‐positive respondents showed that they had tested for HIV, only 50% of malaria‐negative respondents had tested for HIV (*p* < 0.001). Only 17% of malaria‐negative respondents indicated that they knew a place to get tested for HIV (*p* < 0.001). A smaller percentage of malaria‐negative respondents (13.8%) indicated that they would buy food from a vendor if they knew the person had HIV (*p* < 0.001).

**Table 9:** HIV‐positive and malaria‐negative respondents' knowledge about HIV.

Characteristics	Malaria‐negative (*N* = 94)	HIV‐positive (*N* = 103)	*p* value
Knowledge of HIV	87 (92.6%)	103 (100%)	0.004
Having one sex partner reduces the chance of getting HIV	78 (83.0%)	95 (92.2%)	0.003
A condom prevents HIV infection	77 (81.9%)	88 (85.4%)	
HIV can be acquired by sharing food with an infected person	2 (2.1%)	13 (12.6%)	0.002
Tested for HIV	47 (50.0%)	103 (100%)	< 0.001
Know a place to get an HIV test	68 (17.0%)	91 (88.4%)	< 0.001
Will buy food from a vendor if they know the person has HIV	13 (13.8%)	60 (58.3%)	< 0.001

Abbreviation: HIV, human immunodeficiency virus.

## 4 Discussion

This section provides a brief discussion of the major findings from the study on knowledge, attitudes, and practices of malaria and HIV that can be used to create or improve malaria and HIV prevention and control programs. Regarding knowledge, attitude, and prevention of malaria, over 94% of respondents in all categories indicated they knew clinical signs/symptoms, transmission, and prevention of malaria. This is a little higher and almost in agreement with Astatkie [9]. The high level of awareness by the respondents in this study could be attributed to year‐round exposure to mosquito bites and also the Government's struggles to make the general public aware of malaria through television and radio programs, public lectures, and most of all through posters in clinics [3].

It is encouraging that in all categories, most of the households (over 60%) preferred to use mosquito nets to protect their members from malaria. This is in line with Mwanje [13] and Astatkie [9], who reported the use of mosquito nets as the most accepted method of malaria prevention and control by respondents. However, less than 65% of the respondents in all categories showed that they had insecticide‐treated nets in their homes. On the other hand, less than 40% of the respondents in all categories indicated that they slept under insecticide‐treated nets the night before the interview.

Despite the high awareness of ITNs, the relatively low usage rates observed in this study suggest some potential barriers to their adoption. Several of these potential barriers may include access and affordability. Not all individuals may have access to free distributions, and the cost of purchasing ITNs may be prohibitive. Additionally, some individuals may find ITNs uncomfortable due to the heat retention, irritation from insecticides, and the feeling of suffocation while sleeping under them. Misinformation and cultural beliefs may also hinder ITNs usage, with some perceiving it as ineffective while some may associate it with negative superstitions. Household sleeping arrangements, such as open‐air sleeping or multiple people sharing a bed, may also make ITN use impractical. Addressing these barriers through targeted education, improved distribution strategies, and enhanced community engagement is crucial for increasing ITN utilization and ultimately improving malaria control efforts.

The promotion and distribution of insecticide‐treated nets have been going on for years in Ghana. Government, private stakeholders, and nongovernmental organizations have been involved in bed net sourcing and distribution. Therefore, the high level of knowledge of the use of insecticide‐treated nets as a malaria prevention method is not surprising. Given the noticeable interest in mosquito nets as a preventive method against malaria, malaria control programs can build on this prospect to fight malaria. However, it is reasonable to have mass campaigns that distribute free insecticide‐treated nets, but care must be taken to teach people how to properly use them.

Insecticidal spray was indicated as the second (less than 20%) most preferred malaria preventive method by the respondents in all categories. This is high but nearly in accordance with Astatkie [9]. The use of mosquito coil as a malaria preventive method recorded the lowest preference, with less than 7% in all categories. The lower preference for mosquito coils and insecticide spray could be attributed to their associated side effects. Understanding the attitude regarding preferences to intervention measures is essential since noncompliance with adapted strategies on a larger scale could undermine the use and sustainability of such intervention measures aimed at controlling malaria.

Over the years, extensive awareness campaigns on HIV have been conducted locally, and this could have contributed to the increase in HIV knowledge among our participants. The respondents' general knowledge (including mode of transmission and prevention) of HIV was reasonably high (over 94%) in all categories. This is almost in accordance with Ndegwa et al. [4]. A little above 12% of the respondents indicated that HIV can be acquired by sharing food with an infected person. Our study identified some critical misconceptions about HIV transmission. This is in line with Choudhary et al. [14], High [15], Nubed and Akoachere [5], Meena et al. [16] who also reported some misconceptions about the mode of transmission of HIV (e.g., by mosquito bites, sneezing, etc.). These misconceptions are likely to arise from several factors, including a lack of formal education, limited access to accurate health information, deep‐rooted cultural beliefs, and stigma surrounding the disease. In many local communities, myths such as acquiring HIV through casual contact, mosquito bites or sharing food with an infected person persist, contributing to fear and discrimination against persons living with HIV. These fabrications may deter people from seeking testing and treatment, thereby exacerbating the spread of the virus. Again, these misconceptions may result in risky activities such as unprotected sex or having multiple sex partners, which may expose them to infection.

These findings show the need for reinforcement of educational interventions [5], such as community‐based health education programs. Local health workers and religious leaders can be utilized to improve the dissemination of accurate information. In addition, schools should integrate comprehensive sexual health education into their curricula, ensuring young people receive reliable knowledge about HIV early. Also, mass media campaigns using television, radio and social media platforms can help counter myths and reinforce evidence‐based information.

Almost all of the respondents in our study had heard at least one of the HIV preventive methods. Having one sex partner (73.1%, 83%, and 92.2%) and using condoms (79.8%, 81.9%, and 85.4%) as a means of HIV prevention methods were pointed out by respondents. This is consistent with Mohammed [17], Choudhary et al. [14] Oppong and Oti‐Boadi [18], Meena et al. [16].

Voluntary counseling and testing (VCCT) for HIV has been in existence in Ghana for some years now. However, 17% of malaria‐negative respondents indicated they did not know where to get tested for HIV but 74.8% and 88.4% of malaria and HIV‐positive respondents, respectively, showed the opposite. In this study, 50% and 36.7% of malaria‐negative and malaria‐positive respondents, respectively, indicated that they had voluntary counseling and testing for HIV, as against 100% of HIV‐positive persons. This is similar to the report by High [15]. The smaller rate of VCCT could be linked to several barriers, including (1) social stigma and discrimination, which prevent many individuals from seeking VCCT for fear of rejection should their HIV status be exposed; (2) many do not perceive a benefit to knowing their HIV status due to a lack of education regarding available treatment [1, 15]. This suggests a need to encourage the general public about HIV testing to ensure timely and effective treatment for HIV‐positive persons.

One of the major concerns related to HIV is social stigma and discrimination which occurs at individual, family, and societal levels. Stigma and discrimination escalate the HIV epidemic. The whys and wherefores behind these issues are poor information and misconceptions about HIV [16]. In this study, 13.8% and 14.3% of malaria‐negative and malaria‐positive respondents, respectively, indicated significantly that they would not purchase food from a vendor if they knew the person was HIV‐positive. Surprisingly, this response was obtained from 58% of HIV‐positive respondents. This demonstrates that discriminatory attitudes were present in a considerable proportion of the respondents. This level of discrimination is similar to the report by Meena et al. [16] and Nubed and Akoachere [5].

### 4.1 Limitations of the Study

The study design is cross‐sectional and thus limits causal inference. While associations between knowledge, attitude, and practices related to malaria and HIV can be identified, the study cannot establish cause‐and‐effect relationships. Longitudinal studies would be beneficial in assessing changes in knowledge and practices over time, and evaluate the impact of educational interventions. Again, the study relied on self‐reported data, which may be subjected to recall bias. Respondents may have provided answers they believe to be more socially acceptable than their true practices or beliefs, potentially affecting the accuracy of the findings.

## 5 Conclusion

In our cross‐sectional survey, most of the respondents showed that they knew about malaria and HIV. However, some misconception about HIV transmission was observed. Mass media should be utilized in a big way to alleviate the misconceptions associated with HIV within the general population. Community health workers and clinics should provide malaria and HIV education as part of their regular health talks and outreach activities, ensuring that accurate information is consistently available. Counseling of relatives and friends of persons living with HIV should be given the highest priority to prevent stigma and discrimination. The rate of voluntary counseling and testing was low, suggesting a need to encourage the general public about HIV testing to ensure timely and effective treatment for HIV‐positive persons. Mosquito nets were the most preferred preventive method for malaria. During community‐led ITNs distribution programs, local leaders should be involved to ensure effective distribution while educating households on the proper usage of ITNs. Practical demonstration on the correct usage and maintenance of ITNs should be encouraged to improve compliance and sustainability. We also recommend the use of storytelling, peer education and role‐playing techniques to shift attitudes and encourage positive health behaviors.

## Author Contributions

**Felix Abekah Botchway:** conceptualization, writing – original draft, methodology, writing – review and editing, investigation, validation, formal analysis, data curation, software, and visualization. **Cecilia Elorm Lekpor:** writing – review and editing, supervision, conceptualization, methodology, and resources. **Prince Agyeman:** writing – original draft, writing – review and editing. **Ebenezer Krampah Aidoo:** writing – review and editing, and supervision. **Jacob Apibilla Ayembilla:** writing – review and editing, project administration, and supervision. **Micheal Appiah:** project administration and supervision. **Ahmed Mashud:** supervision and project administration. **Richard Kobina Dadzie Ephraim:** writing – review and editing and project administration.

## Ethics Statement

Ethical clearance was obtained from the Ethical and Protocol Review Committee of Accra Technical University ATU/MLT/ET/401/2022. Participants were informed about the purpose of the study, and written informed consent was obtained before data collection. Confidentiality and anonymity were strictly maintained. Special care was taken to ensure that respondents from vulnerable rural populations were not coerced into participation, and they were assured of their right to withdraw from the study at any time without consequences. Additionally, measures were implemented to protect sensitive information, particularly regarding the status of HIV, to prevent potential stigmatization and discrimination.

## Conflicts of Interest

The authors declare no conflicts of interest.

## Transparency Statement

The lead author Felix Abekah Botchway affirms that this manuscript is an honest, accurate, and transparent account of the study being reported; that no important aspects of the study have been omitted; and that any discrepancies from the study as planned (and, if relevant, registered) have been explained.

## Data Availability

The data that support the findings of this study are available from the corresponding author upon reasonable request. The data used in this study are not publicly available due to confidentiality of information; however, the data is available from the corresponding author on a reasonable request basis.
